# Utility of urinary biomarkers in primary haematuria: Systematic review and meta‐analysis

**DOI:** 10.1002/bco2.147

**Published:** 2022-03-28

**Authors:** Nicolas Adrianto Soputro, Dylan Neil Gracias, Brendan Hermenigildo Dias, Tatenda Nzenza, Helen O'Connell, Kapil Sethi

**Affiliations:** ^1^ Department of Urology Western Health Footscray Victoria 3011 Australia; ^2^ Melbourne Medical School, Faculty of Medicine, Dentistry, and Health Sciences University of Melbourne Parkville Victoria 3010 Australia; ^3^ Department of Surgery The University of Melbourne Parkville Victoria 3010 Australia

**Keywords:** biomarker, bladder cancer, haematuria

## Abstract

**Objectives:**

To evaluate the diagnostic performance of FDA‐approved urinary biomarkers in the evaluation of primary haematuria for investigation of bladder cancer.

**Methods:**

The scientific databases MEDLINE, EMBASE, Pubmed and Web of Science were searched to collect studies. Studies that evaluated the diagnostic performance of FDA‐approved urinary biomarkers in investigating patients with primary haematuria without a prior history of bladder cancer were included. Quality of studies was assessed using the JBI Criteria. Bivariate mixed‐effects regression model was used to calculate pooled sensitivities and specificities for each biomarker.

**Results:**

Eighteen studies were included in the analysis. The biomarkers assessed in these studies were CxBladder, AssureMDx, Bladder Tumour Antigen (BTA), NMP22, UroVysion and Immunocyt/uCyt+. Several biomarkers, such as AssureMDx, CxBladder and Immunocyt, were shown to have better diagnostic performance based on their sensitivity, specificity and diagnostic odds ratio, as well as positive and negative likelihood ratios. Across the six biomarkers, sensitivity ranged from 0.659 to 0.973, and the specificity ranged between 0.577 and 0.833.

**Conclusion:**

Despite certain biomarkers demonstrated better performance, current diagnostic abilities of the FDA‐approved biomarkers remain insufficient for their general application as a rule out test for bladder cancer diagnosis and as a triage test for cystoscopy in patients with primary haematuria. High‐quality prospective studies are required to further analyse this and also analyse the correct scenario in which urinary biomarkers may be best utilised.

## 1 INTRODUCTION

Bladder cancer is the ninth most common cancer in the world, with a steadily rising incidence, especially in developing countries and resource‐poor settings.^1^, ^2^ It accounts for 3% of global cancer diagnosis and more typically affecting males aged 55 years and older. Patients typically present with painless macroscopic haematuria, which often prompts clinicians to specifically investigate for urothelial carcinoma by means of urine cytology, urographic computed tomography (CT), and cystoscopy. Both CT urogram and cystoscopy are known to have high sensitivities of up to 82% and 100% and high specificities of up to 94% and 97%, respectively.^3^ Recent technological developments, such as the introduction of narrow‐band imaging and blue‐light cystoscopy, have further improved diagnostic capacity of cystoscopy, especially for non‐invasive lesions and carcinoma in situ. At present, urine cytology remains the most routinely used urinary marker to investigate for malignancy. However, it has been attributed to low sensitivities of 34–55% with even reduced sensitivity in low grade tumours and considerable inter‐ and intra‐observer variabilities, despite relatively high specificity of approximately 90%.^4^

Haematuria itself is a common clinical presentation, occurring in up to 9–18% of the population. It is not pathognomonic for bladder cancer with an approximately 12% patients being investigated for haematuria confirmed to have bladder cancer.^5^ This highlights significant issues about how patients should be evaluated as current investigations may be invasive, uncomfortable and morbid, all whilst failing to produce a satisfactory diagnostic yield. One approach is to utilise the diagnostic capacity of urinary markers to determine when to perform cystoscopy. Recent years have seen the introduction of these urinary biomarkers with some already approved by Food and Drug Administration (FDA). These include AssureMDx (MDxHealth, Irvine, CA, USA), BTA (Polymedco, Cortlandt, NY, USA), CxBladder (Pacific Edge Ltd., Dunedin, New Zealand), NMP22 (Matritech Inc., Newton, MA, USA), UroVysion (Vysis, Abott Molecular Inc., IL, USA), and uCyt+ assay (Scimedx Inc., Denville, NJ, USA).^6^, ^7^ Limited studies have been performed to objectively compare the efficacy of these biomarkers in macroscopic haematuria. This meta‐analysis therefore aims to assess the performance of these FDA‐approved urinary biomarkers in the identification of bladder cancer in primary macroscopic haematuria.

## 2 METHOD

### 2.1 FDA‐approved biomarkers

uCyt+/Immunocyt is a commercially available assay utilising immunocytological technology to detect tumour‐associated antigens in urothelial cells. As per the manufacturer's instructions, sample urine is prepared with three fluorescein‐labelled monoclonal antibodies which have affinity to particular tumour associated antigens. These slides are subsequently examined microscopically for immunofluorescence. Specimens with more than one green or red urothelial cell are considered to be positive.^8^, ^9^, ^10^, ^11^

The bladder tumour antigen (BTA) test employs monoclonal antibodies to detect elevated levels of complement factor H‐related protein (CFHrp) in voided urine, which is a degradation product of the basement membrane shown to be released by malignant cells in culture. There are two types of BTA tests, including the qualitative BTA Stat and the quantitative BTA Trak, which is an enzyme‐linked immunosorbent assay.^12^, ^13^, ^14^, ^15^

Nuclear matrix proteins (NMP) are a group of proteins which provide a structural framework to the nucleus and are involved in DNA replication and RNA synthesis. The NMP22 test in particular detects nuclear mitotic apparatus protein which has been shown to be more abundant in malignant urothelial cells. Upon apoptosis, nuclear mitotic apparatus proteins can be detected in the urine at significantly elevated levels than normal. There are two tests to detect NMP22 levels, including the original quantitative sandwich type immunoassay and the qualitative BladderChek test.^11^, ^12^, ^16^, ^17^, ^18^, ^19^, ^20^, ^21^

AssureMDx isolates DNA from urine samples and analyses these for three mutation genes (FGFR3, TERT and HRAS) and three methylation genes (OTX1, ONECUT2 and TWIST1).^22^

UroVysion is another fluorescence‐based assay that uses fluorescence in situ hybridisation (FISH) to observe multiple different chromosomal copy numbers and DNA sequences in cell nuclei derived from a urine sample. Various genetic alterations are examined for in this test, including aneuploidy of chromosomes 3, 7 and 17, and the loss of the 9p21 locus, which are 4 chromosomal changes frequently associated with urothelial carcinoma.^11^, ^23^

CxBladder extracts and quantifies five mRNA biomarkers (MDA, HOCXA13, CDC2, IGFBP5 and CXCR2) known to be differentially expressed in malignant cells than in normal cells. These biomarkers are quantified in urine samples using reverse transcriptase quantification polymerase chain reaction.^24^

### 2.2 Search strategy

The Preferred Reporting Items for Systematic Reviews and Meta‐Analysis (PRISMA) framework was implemented for this review with search performed in medical literature databases, including EMBASE, MEDLINE, PubMed, and Web of Science, accessible to The University of Melbourne library, St. Vincent's Hospital Melbourne library and Western Health library. Keywords used included “bladder cancer,” “bladder carcinoma,” “bladder malignancy,” “bladder neoplasm,” “bladder tumour,” “bladder tumor,” “haematuria,” “hematuria,” “biomarker” or “urinary biomarker,” including for each of the FDA‐approved biomarkers separately. Boolean operators were utilised to combine the sets of searches. Separate searches were performed by two independent authors (N.S. and D.G.) performing title and abstract screening independent of each other according to our inclusion and exclusion criteria. After collating search results and removing duplicates from the respective searches, full texts of the relevant articles were then reviewed for their quality using the Joanna Briggs Institute (JBI) Criteria for Diagnostic Test Accuracy Studies. Full text articles that were unable to be accessed or obtained through our institutions were excluded. Where necessary, disagreements were resolved in consultation with a senior author (K.S.).

### 2.3 Inclusion and exclusion criteria

Prospective studies written in English and performed on adult patients of 18 years of age and above who presented with primary macroscopic haematuria without prior diagnosis of bladder cancer were included in our analysis. Patients in the studies needed to be tested with at least one of the FDA‐approved biomarkers in addition to cystoscopy, either rigid or flexible. Prospective and retrospective studies were included. No restrictions on date of publications were imposed on the studies. Studies whose patients presented with microscopic haematuria, demonstrated recurrence of bladder cancer, and those without any follow‐up cystoscopy were excluded.

### 2.4 Statistical analysis

For each included study in the meta‐analysis, Sensitivity, specificity, positive and negative predictive values were calculated. Pooled sensitivities and specificities and subsequent comparisons of biomarkers were calculated using a bivariate random‐effects regression model of meta‐analysis as described by Reitsma et al. and Harbord et al. using the restricted maximum likelihood (REML) method of estimating variance.^25^, ^26^ Statistical analysis was performed in R (version 3.4, R foundation for statistical Computing, Vienna, Austria). The “mada” package in R was used to design forest plots for pooled specificity, sensitivity and diagnostic odds ratios based on individual biomarkers and individual studies. The analysis was plotted using summary receiver operating characteristics (SROC) curves in R.^27^

## 3 RESULTS

**FIGURE 1:**
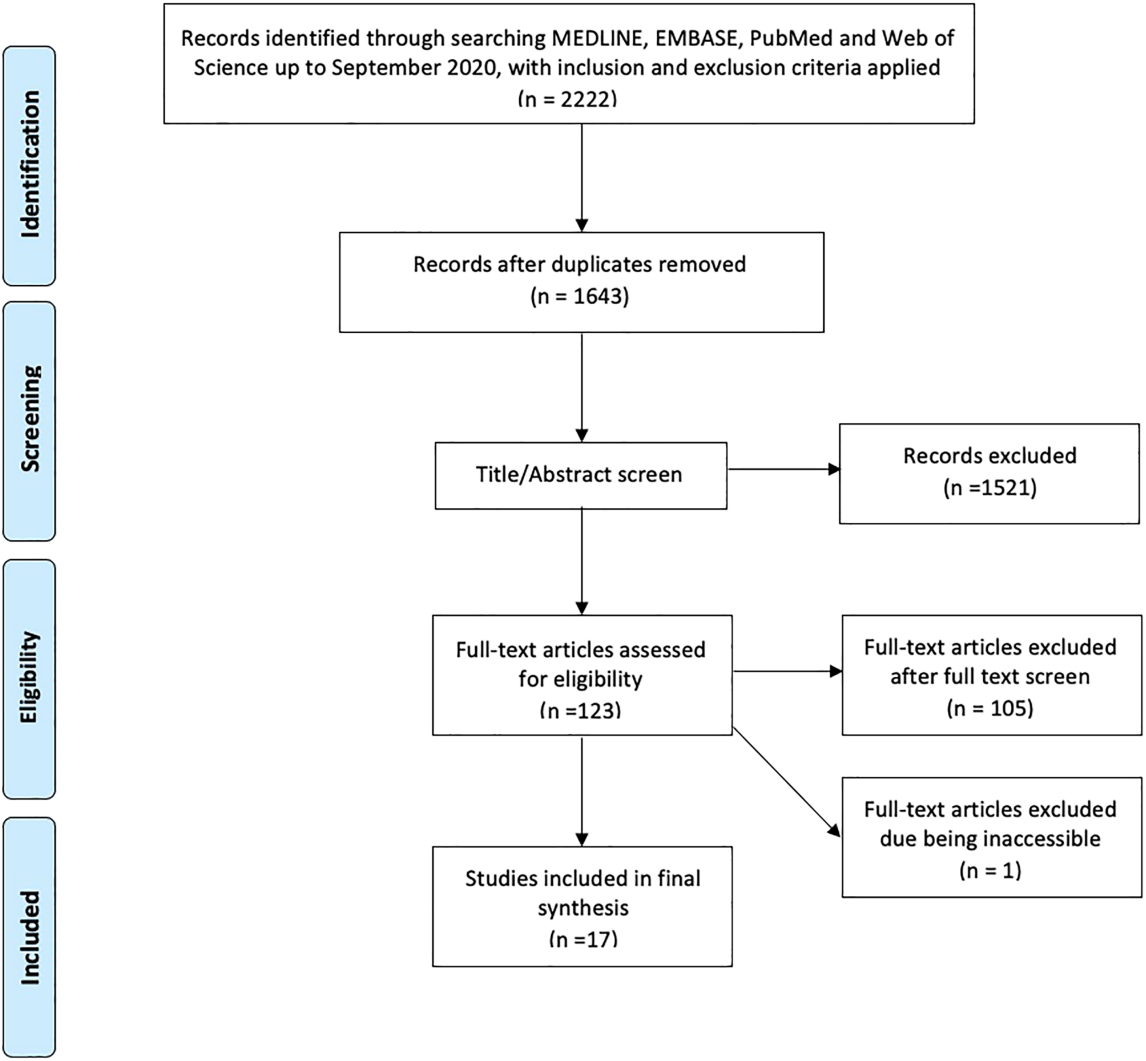
PRISMA flow chart

A flowchart of our search based on the PRISMA framework was as depicted in Figure 1. Our initial search of the literature on available database yielded 2222 titles/abstracts review, from which 579 were duplicate records and hence were excluded. Following title and abstract screen from 1643 records, 123 articles were selected for full‐text review. Unfortunately, one was not accessible to facilitate full‐text review and was excluded.^28^ Seventeen articles met our inclusion criteria,^8^, ^9^, ^10^, ^11^, ^12^, ^13^, ^14^, ^15^, ^16^, ^17^, ^18^, ^19^, ^20^, ^21^, ^23^, ^24^ where results pertaining to each biomarker were extracted with their characteristics were as presented in Table 1 and the calculated sensitivity, specificity and diagnostic odds ratio, as well as the positive and negative likelihood ratio for each of the extracted biomarker as shown in Table 2.

**TABLE 1:** Characteristics of included studies separated into the different urinary markers each with the calculated sensitivity, specificity, positive predictive value (PPV) and negative predictive value (NPV)

Author	Year	Biomarker	Sample size	Age^a^ median (range)	Number of cancers diagnosed (%)	Sensitivity	Specificity	PPV	NPV
Van Kessel et al.^22^	2017	AssureMDx	154	(21–91)	74 (48%)	97%	83%	23.40%	99.10%
Abogunrin et al.^12^	2012	BTA	156	NR	79 (51%)	73%	68%		
Efthimiou et al.^14^	2011	BTA	122	(40–88)	34 (28%)	47%	72.70%	40.00%	78.00%
Chong et al.^13^	1999	BTA	47	(28–86)	12 (26%)	67%	66%	40%	85%
Kirollos et al.^15^	1997	BTA	24	67	1 (4%)	100%	73.90%	14.30%	100%
O'Sullivan et al.^24^	2012	CxBladder	485	69 (45–80)	66 (14%)	81.80%	85.10%	46.60%	96.70%
Deininger et al.^9^	2017	Immunocyt	444	67 (20–93)	68 (15%)	86.80%	78.70%	42.40%	97%
Todenhofer et al.^11^	2012	Immunocyt	449	65.5 (18–93)	67 (15%)	88.10%	80.10%	44.40%	97.40%
Cha et al.^8^	2012	Immunocyt	1182	65 (18–93)	245 (21%)	82.40%	86.60%	61.60%	95%
Schmitz‐Drager et al.^10^	2008	Immunocyt	63	(24–89)	17 (27%)	88.20%	80.40%	62.50%	94.90%
Abogunrin et al.^12^	2012	NMP22	104	NR	65 (63%)	60%	87%	88.60%	56.70%
Todenhofer et al.^11^	2012	NMP22	449	65.5 (18–93)	67 (15%)	89.40%	34.10%	19.30%	94.80%
Srirangam et al.^20^	2011	NMP22	162	(33–89)	110 (68%)	62.70%	86.50%	90.80%	52.30%
Lotan et al.^16^	2010	NMP22	206	59 (18–96)	38 (18%)	68.40%	79.20%	42.60%	91.70%
Talwar et al.^21^	2007	NMP22	69	(39–78)	12 (17%)	91.70%	91.20%	68.80%	98.10%
Sawczuk et al.^19^	2005	NMP22	56	(19–93)	21 (38%)	71%	74%	63%	81%
Oge et al.^17^	2001	NMP22	37	(26–87)	27 (73%)	74.10%	60.00%	83.30%	46.20%
Paoluzzi et al.^18^	1999	NMP22	90	NR	32 (36%)	84%	62%	30%	40%
Todenhofer et al.^11^	2012	Urovysion	449	65.5 (18–93)	67 (15%)	72.70%	86.30%	49%	94.60%
Sarosdy et al.^23^	2006	Urovysion	473	(40–97)	51 (11%)	68.60%	77.70%	27.10%	95.30%

Abbreviations: BTA, bladder tumour antigen; NMP22, nuclear matrix protein 22.

a Age presented in years.

**TABLE 2:** Sensitivity, specificity, diagnostic odds ratio, positive likelihood ratio, and negative likelihood ratio analysed for each urinary biomarker extracted from the included studies

Author	Year	Biomarker	Sensitivity (95% CI)	Specificity (95% CI)	Diagnostic Odds Ratio	Positive Likelihood Ratio	Negative Likelihood Ratio
Van Kessel et al.^22^	2017	AssureMDx	0.967 (0.898–0.990)	0.821 (0.724–0.889)	133	5.4	0.041
Abogunrin et al.^12^	2012	BTA	0.598 (0.478–0.708)	0.862 (0.724–0.938)	9.350	4.353	0.466
Efthimiou et al.^14^	2011	BTA	0.471 (0.317–0.631)	0.725 (0.624–0.807)	2.348	1.713	0.729
Chong et al.^13^	1999	BTA	0.654 (0.389–0.849)	0.653 (0.489–0.787)	3.551	1.883	0.530
Kirollos et al.^15^	1997	BTA	0.750 (0.198–0.973)	0.729 (0.529–0.866)	8.077	2.769	0.343
O'Sullivan et al.^24^	2012	CxBladder	0.813 (0.704–0.889)	0.851 (0.714–0.882)	24.939	5.466	0.219
Deininger et al.^9^	2017	Immunocyt	0.862 (0.762–0.925)	0.786 (0.742–0.825)	23.069	4.038	0.175
Todenhofer et al.^11^	2012	Immunocyt	0.890 (0.793–0.944)	0.341 (0.295–0.390)	4.169	1.350	0.324
Cha et al.^8^	2012	Immunocyt	0.823 (0.771–0.866)	0.865 (0.842–0.886)	29.863	6.104	0.204
Schmitz‐Drager et al.^10^	2008	Immunocyt	0.861 (0.639–0.956)	0.798 (0.663–0.888)	24.474	4.260	0.174
Abogunrin et al.^12^	2012	NMP22	0.731 (0.625–0.816)	0.673 (0.563–0.767)	5.602	2.237	0.399
Todenhofer et al.^11^	2012	NMP22	0.728 (0.612–0.819)	0.863 (0.825–0.894)	16.844	5.311	0.315
Srirangam et al.^20^	2011	NMP22	0.626 (0.533–0.710)	0.858 (0.740–0.928)	10.160	4.425	0.436
Lotan et al.^16^	2010	NMP22	0.679 (0.523–0.804)	0.790 (0.722–0.845)	7.972	3.235	0.406
Talwar et al.^21^	2007	NMP22	0.885 (0.621–0.973)	0.905 (0.803–0.957)	73.182	9.329	0.127
Sawczuk et al.^19^	2005	NMP22	0.705 (0.496–0.853)	0.736 (0.575–0.852)	6.652	2.670	0.401
Oge et al.^17^	2001	NMP22	0.732 (0.548–0.860)	0.591 (0.316–0.819)	3.948	1.790	0.453
Paoluzzi et al.^18^	1999	NMP22	0.833 (0.673–0.924)	0.619 (0.491–0.732)	8.111	2.185	0.269
Todenhofer et al.^11^	2012	Urovysion	0.875 (0.776–0.934)	0.800 (0.757–0.837)	28.046	4.381	0.156
Sarosdy et al.^23^	2006	Urovysion	0.683 (0.547–0.793)	0.777 (0.735–0.814)	7.479	3.056	0.409

Abbreviations: BTA, bladder tumour antigen; NMP22, nuclear matrix protein 22.

**FIGURE 2:**
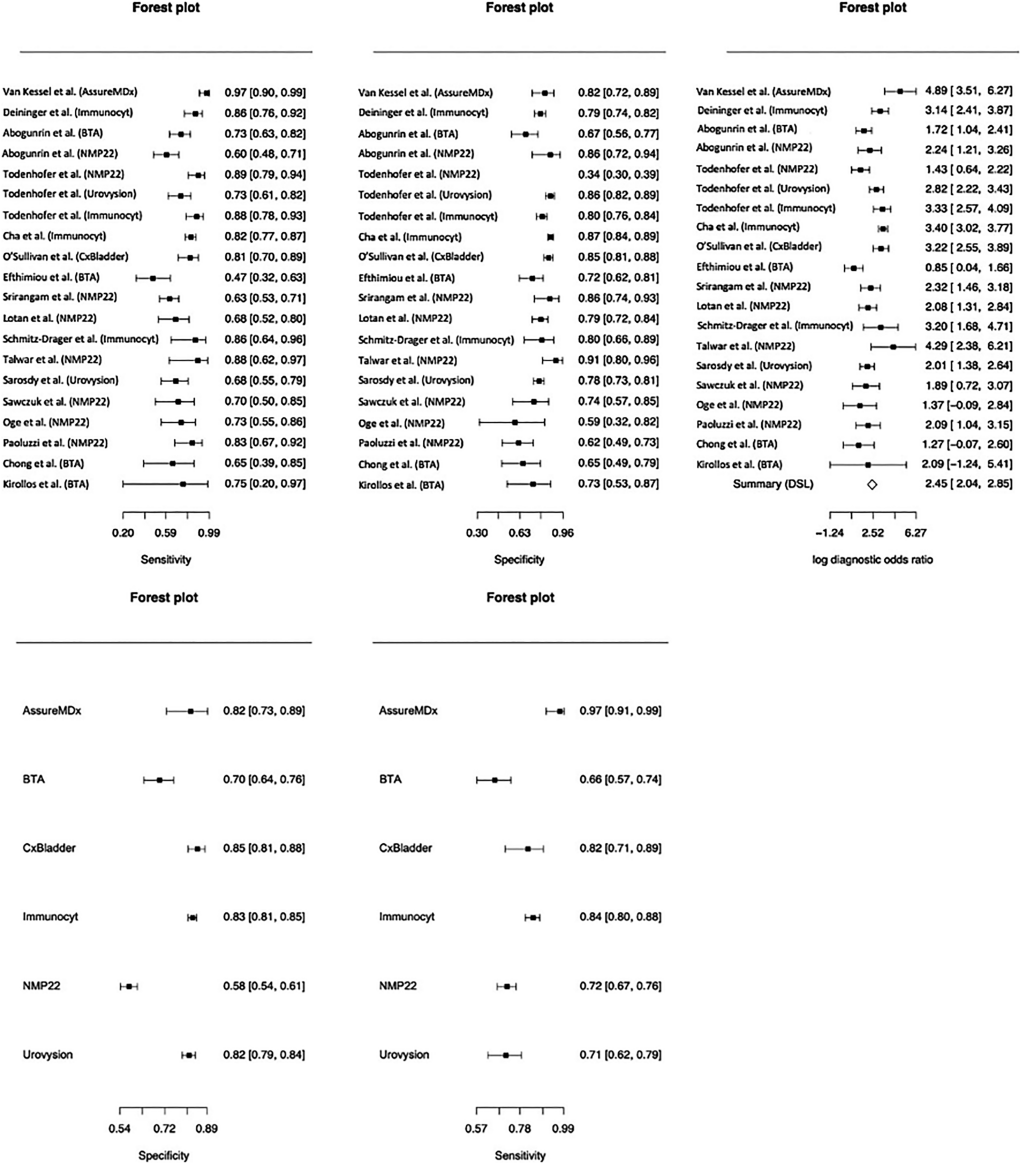
Sensitivity, specificity and diagnostic odds ratio of each biomarker from the individual studies as well as pooled sensitivity and specificity of the different FDA‐approved biomarkers

Compared with other biomarkers, NMP22 was discussed in most with eight studies,^11^, ^12^, ^16^, ^17^, ^18^, ^19^, ^20^, ^21^ followed by BTA^12^, ^13^, ^14^, ^15^ and Immunocyt^8^, ^9^, ^10^, ^11^ in four studies each, CxBladder^24^ and UroVysion^11^, ^23^ in two studies each, and AssureMDx in one study. Where reported, the age of patients included ranged from 18 to 97 years, with medians ranging from 59 to 69 years. The number of patients included with histopathological diagnosis of bladder cancer varied from one to 245 patients, which comprised 4–73% of the respective recruited cohorts. The sensitivities, specificities, PPV and NPV were as presented in Table 1. The performance of each biomarker based on the individual studies were as summarised in Figure 2. Where results were not reported in the respective study, calculations were performed to determine the sensitivities, specificities, PPV and NPV.

We identified one study that assessed diagnostic performance of AssureMDx for primary haematuria^22^ with sensitivity of 0.973 (95% CI 0.907–0.993) and specificity of 0.825 (95% CI 0.727–0.893). The calculated diagnostics odds ratio (DOR), positive likelihood ratio (LR), and negative LR were 169.714 (37.164–775.022), 5.560 (3.450–8.961) and 0.033 (0.008–0.129). Compared with other urinary markers, AssureMDx was noted to be the best performing with highest sensitivity, as well as highest positive LR and lowest negative LR.

Similarly, the performance of CxBladder was only based on one study^24^ with a sensitivity and specificity of 0.818 (95% CI 0.709–0.893) and 0.852 (95% CI 0.815–0.883), respectively. The calculated DOR, positive LR and negative LR were 25.911 (13.112–51.205), 5.529 (4.279–7.145) and 0.213 (0.128–0.357), respectively.

Four studies evaluated the use of BTA.^12^, ^13^, ^14^, ^15^ The pooled sensitivity and specificity were 0.659 (95% CI 0.572–0.736) and 0.7 (95% CI 0.636–0.756), respectively. The summary of the DOR, positive LR and negative LR from the four studies were 4.494 (2.819–7.165), 2.192 (1.731–2.777) and 0.488 (0.377–0.631).

Four studies relating to Immunocyt met our inclusion criteria.^8^, ^9^, ^10^, ^11^ The pooled sensitivity from the four studies was 0.844 (95% CI 0.805–0.876), and the pooled specificity was 0.833 (95% CI 0.815–0.850). The calculated DOR, positive LR and negative LR were 26.923 (19.969–36.299), 5.048 (4.509–5.653) and 0.188 (0.149–0.236), respectively.

Most of the studies included in this review relate to the use of NMP22 for evaluation of patients with primary macroscopic haematuria.^11^, ^12^, ^16^, ^17^, ^18^, ^19^, ^20^, ^21^ The pooled sensitivity from the eight studies was 0.718 (95% CI 0.670–0.761), and the pooled specificity was 0.577 (95% CI 0.542–0.611). We calculated the DOR to be 3.465 (2.657–4.520), positive LR of 1.696 (1.530–1.880) and negative LR of 0.489 (0.412–0.582).

Two studies pertaining to the use of Urovysion for investigation of primary haematuria were included^11^, ^23^ from which the pooled sensitivity and specificity were 0.712 (95% CI 0.624–0.786) and 0.818 (95% CI 0.790–0.844). The summary of analysed DOR, positive LR and negative NR were 11.135 (7.193–17.235), 3.920 (3.254–4.723) and 0.352 (0.265–0.468), respectively.

**FIGURE 3:**
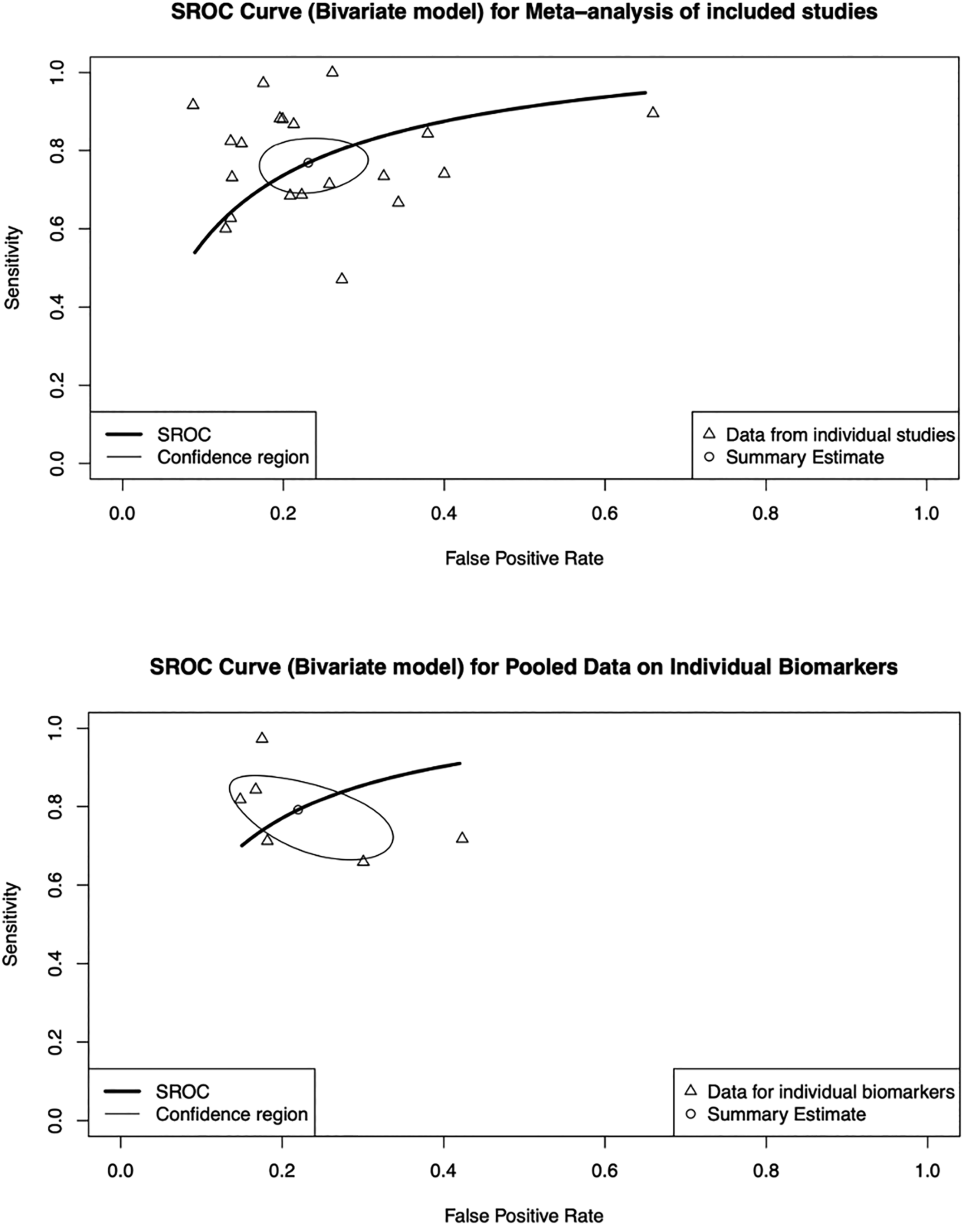
SROC curves for each biomarker based on the individual studies (top) and pooled estimates (bottom)

We used a bivariate diagnostic random‐effects model of meta‐analysis using the REML method of estimating variance. The SROC plots for the included studies is shown in Figure 3. Using this model, the sensitivity, false positive rate and area under the curve (AUC) was 0.769 (95% CI 0.707–0.821), 0.231 (95% CI 0.181–0.290) and 0.834. We also used a similar model to plot SROC curve for the pooled data for the different biomarkers as also shown in Figure 3. Similarly, the sensitivity, false positive rate and AUC of the model were 0.785 (95% CI: 0.706–0.848), 0.220 (95% CI: 0.154–0.304) and 0.849, respectively.

## 4 DISCUSSION

The findings from our meta‐analysis demonstrated the sensitivities of the FDA‐approved biomarkers to range between 0.659 and 0.973 and their specificities to range between 0.577 and 0.833, as highlighted in Figure 2 and Table 3. These results were consistent with reported sensitivities of 0.67–0.95 and specificity of 0.68–0.87.^6^ Routine work‐up of patients presenting with haematuria typically consists of cystoscopy, radiological imaging and urine cytology. Of these, cystoscopy remains as the gold standard for diagnosis of bladder cancer. A systematic review analysing the diagnostic accuracy of cystoscopy demonstrated a sensitivity and specificity ranging from 68.3–100% and 57–97%, respectively. This was dependent on the different types of cystoscopic adjuncts applied such as blue light and narrow band imaging, which were shown to further improve diagnostic accuracy.^3^ Despite the advances, the complication profiles including for discomfort, invasive nature, urinary tract infection, and lower urinary tract symptoms, such as frequency, dysuria, and haematuria, may not support the argument towards the regularity of its use.^29^ Hence, the inclusion of urine cytology and urinary markers in the diagnostic algorithm may serve as a triage test for higher risk patients to proceed for cystoscopy.

**TABLE 3:** Calculated pooled sensitivity, specificity, diagnostic odds ratio, positive likelihood ratio, and negative likelihood ratio analysed for the six FDA‐approved biomarkers

Biomarker	Number of Studies	Sensitivity (95% CI)	Specificity (95% CI)	Diagnostics odds ratio (DOR)	Positive likelihood ratio	Negative likelihood ratio
AssureMDx	1	0.973 (0.907–0.993)	0.825 (0.727–0.893)	169.714 (37.164–775.022)	5.560 (3.450–8.961)	0.033 (0.008–0.129)
BTA	4	0.659 (0.572–0.736)	0.7 (0.636–0.756)	4.494 (2.819–7.165)	2.192 (1.731–2.777)	0.48 (0.377–0.631)
CxBladder	1	0.818 (0.709–0.893)	0.852 (0.815–0.883)	25.911 (13.112–51.205)	5.529 (4.279–7.145)	0.213 (0.128–0.357)
Immunocyt	4	0.844 (0.805–0.876)	0.833 (0.815–0.850)	26.923 (19.969–36.299)	5.048 (4.509–5.653)	0.188 (0.149–0.236)
NMP22	8	0.718 (0.670–0.761)	0.577 (0.542–0.611)	3.465 (2.657–4.520)	1.696 (1.530–1.880)	0.489 (0.412–0.582)
Urovysion	2	0.712 (0.624–0.786)	0.818 (0.790–0.844)	11.135 (7.193–17.235)	3.920 (3.254–4.723)	0.352 (0.265–0.468)

Abbreviations: BTA, bladder tumour antigen; NMP22, nuclear matrix protein 22.

Current international guidelines, such as one by The American Urological Association (AUA) do not recommend the use of urine cytology and biomarkers, specifically NMP22, the qualitative BTA‐STAT, and Urovysion for both macroscopic and microscopic haematuria, the latter defined as >3 red blood cells per high power field in a sample. Other guidelines, like one established by the European Association of Urology (EAU) and Canadian Consensus Document favoured the inclusion of urine cytology but not urinary markers.^30^ This is due to the inadequate and inconsistent performance characteristics, as well as the high false positive results. Urine cytology itself involves the identification of abnormal cytological appearance associated with tumour cells on microscopy. The sensitivity and specificity of urine cytology was previously reported to range between 10.3–84.6% and 78–100%, respectively, with efficacy better for higher grade tumours and carcinoma in situ.^31^ This is due to more well‐differentiated tumours being less likely to shed into the urine and hence less likely to be identified during fixation and microscopy. Certain factors that can influence false positive results include diurnal variations with morning urine more likely to be associated with cytolysis, stone disease, inflammation and infection, presence of proteinuria, as well as radiation changes. In addition, intra‐ and inter‐observer variability impacting on reproducibility of the results are important to acknowledge.^10^, ^11^, ^32^, ^33^ Several studies included in our meta‐analysis also reviewed the performance of urine cytology, which again demonstrated the good specificity ranging from 0.945 to 1 but with sensitivity between 0.211 to 0.66.^10^, ^13^, ^15^, ^18^, ^19^, ^20^, ^21^, ^24^ When compared between different tumour grades, Sawczuk et al. identified better performance of urine cytology in higher grades of bladder cancer with none of the 6 and 10 patients with Grade I and II tumours identified, respectively, whilst one each of the 3 Grade III and 2 Grade IV patients were diagnosed.^19^ Similar findings were reported in a later study by Srirangam et al. with 75% patients with Grade III tumours or carcinoma in situ detected with urine cytology, compared with 0 and 39.5% for Grade I and II, respectively.^20^

The result from our meta‐analysis demonstrated AssureMDx to perform better than other biomarkers, achieving the highest sensitivity of 97.3% and the second highest specificity of 82.5%, after Immunocyt at 83.3%. This biomarker also had the highest diagnostic odds ratio and the lowest negative likelihood ratio hence opening the possibility of AssureMDx as a future triage test.^22^ The superiority of this biomarker compared to others may stem from its design of combining genomic alteration, such as in FGFR3 gene, which subsequently encodes a tyrosine kinase receptor that can activate the often‐dysregulated Ras‐MAPK pathway in bladder cancer, with distinct DNA methylation patterns that can affect different processes, ranging from cell cycle arrest, transcription, apoptosis, and cell differentiations.^34^ However, despite our meta‐analysis outlined the different performance between the biomarkers, there were limited studies performing head‐to‐head comparison between the urinary markers within the same cohort of patients. Of which, Abogunrin et al. attempted to establish the different performance of several biomarkers based on 80 patients with histopathological diagnosis of urothelial carcinoma, including for BTA and NMP22 where they identified sensitivities of 0.73 versus 0.60 and specificities of 0.68 versus 0.87. NMP22 was shown to enhance the diagnostic algorithm, including for clinical factors such as age and smoking history.^12^ Todenhofer et al. also took on NMP22 and compared its performance with Immunocyt based on 67 bladder cancer patients. The summarised sensitivity and specificity were 88.1% versus 89.4% and 80.1% versus 34.1% for Immunocyt and NMP22, respectively.^11^

Similar to urine cytology, it is important to note the performance of the urinary markers in different stages and grades of bladder cancer. Van Kessel et al., for example, presented the improved performance of AssureMDx in stages T1 and above, compared with Ta.^22^ Cha et al. also presented similar phenomenon for Immunocyt with 100% of pT3 and pT4 tumours were positive for the biomarker compared to the 63.7%, 6%, 18.9%, and 8.5% for pTa, pTis, pT1 and pT2, respectively.^8^ Comparable findings were reported for NMP22 with improved sensitivity and specificity for higher grade tumours.^17^, ^18^, ^19^, ^20^, ^21^ Öge et al. presented an improved sensitivity from 55% for pTa tumours to 83% for pT1 and pT2 tumours.^17^ Similarly, Sawczuk et al. reported 100% detection rate of tumours of stages pT1 and above, compared to only 60% for pTa tumours.^19^ Furthermore, Paoluzzi et al. also highlighted the different median NMP22 values for each grade with 35 U/ml for Grade 0, 30 U/ml for Grade 1, 66 U/ml for Grade 2, 54 U/ml for Grade 3 and 102 U/ml for carcinoma in situ. These were compared with the lower median value of 19.1 U/ml for patients without malignancy and well above the commonly used cut‐off of 10 U/ml for NMP22.^18^ In addition to tumour stages and grades, several factors can influence the results of these urinary markers. Similar to urine cytology, increased risk of false positive reading can be associated with urinary tract infection, calculous disease, prostatic hyperplasia and previous instrumentations. Other factor may also have different impacts on each of the urinary marker. For example, increasing creatinine appeared to improve the sensitivity of CxBladder but demonstrated no impacts for NMP22 and UroVysion. Todenhofer et al. showed the presence of urinary protein of greater than 149 mg/dl to increase the false positive rate of NMP22 from 30.7% to 66.7%, but without proven effect on UroVysion and CxBladder.^11^, ^24^

Although this meta‐analysis highlighted the different efficacy of FDA‐approved biomarkers in the evaluation of primary haematuria for bladder cancer, several limitations persist. Firstly, some urinary marker results were based on limited number of studies, such as AssureMDx solely based on study by Van Kessel et al., as well as CxBladder and UroVysion each based on two studies.^11^, ^22^, ^24^ Sample size of most studies were also limited and varying, ranging from only one histologically proven bladder cancer to 245. Thus, one might question the reproducibility of the results in larger studies with potentially more heterogenous cohort. Moreover, despite most diagnostic algorithms involved the use of cystoscopy and histopathological confirmation shortly following sample collection and up to 3 months, there may appear to be some heterogeneity in the cystoscopic evaluations that is dependent on technique, imaging modality, and interobserver variability.^11^, ^14^, ^16^, ^24^

Furthermore, given current evidence that bladder cancer more likely to present with gross haematuria and that microscopic haematuria more likely to be attributed to false positive results,^19^, ^24^ our analysis only included a sub‐group of bladder cancer patients presenting with primary macrohaematuria. However, a different finding was reported by Cha et al. which identified similar performance of Immunocyt in patients presenting with microscopic versus macroscopic haematuria, with sensitivity of 81% versus 84%, and specificity of 88% versus 84%, respectively.^8^ Further research will be required to establish the efficacy of the different biomarkers in setting of micro‐ and macroscopic haematuria for work‐up of bladder cancer.

This review demonstrated the differing performance of six FDA‐approved urinary biomarkers. There remains a paucity of evidence to allow general application as a rule out test in the diagnosis of bladder cancer. Further research is required to provide better clarity on diagnostic performance amongst various stages and grades of bladder cancer detection and to assess if their performance can be consistently close to that of cystoscopy as the current gold standard prior to considering their inclusion in future guidelines for haematuria work‐up. Despite some biomarkers, such as AssureMDx, Immunocyt and CxBladder, showed promising results, we still recommend against their routine use as a triage test for cystoscopy in patients with primary gross haematuria. With available studies still reliant on the use of cystoscopy, future studies may need to consider selecting for a subgroup of patients where the use of these biomarkers as a screening tool for patients with low priority for cystoscopy can be better evaluated.

## AUTHOR CONTRIBUTIONS

NAS was responsible for performing the literature search, write‐up and editing of manuscript; DNG was responsible for performing the literature search and write‐up of the manuscript; BHD was responsible for write‐up and editing of manuscript; TZ, HO and KS were responsible for conceptualisation and editing.
